# Altered microRNAs expression in T cells of patients with SLE involved in the lack of vitamin D

**DOI:** 10.18632/oncotarget.19062

**Published:** 2017-07-07

**Authors:** Dao-Jun Chen, Lan-Ju Li, Xiao-Ke Yang, Tao Yu, Rui-Xue Leng, Hai-Feng Pan, Dong-Qing Ye

**Affiliations:** ^1^ Department of Epidemiology and Biostatistics, School of Public Health, Anhui Medical University, Hefei, Anhui, China; ^2^ Anhui Province Key Laboratory of Major Autoimmune Diseases, Hefei, Anhui, China

**Keywords:** microRNA, vitamin D, SLE, T cells

## Abstract

Vitamin D has been recognized as a potent immunomodulator and its deficiency is common in different population groups including patients with SLE. As miRNAs regulation plays a significant role in SLE, the present study aimed to evaluate the association between vitamin D status and miRNAs levels in patients with SLE. The serum concentrations of vitamin D (25-hydroxyvitamin D) and the levels of six miRNAs in T cells from patients with SLE were measured in 42 SLE cases and 48 healthy controls. Vitamin D treatment was also performed in isolated and cultured T cells from SLE patients in different times and doses. Vitamin D insufficiency (25-hydroxyvitamin D concentration <20 ng/ml) was more common in cases than in controls. Although age and BMI were similar, cases had significantly lower concentrations of miRNA-377, miRNA-342, miRNA-10a, miRNA-374b, miRNA-125a, and miRNA-410 than controls. Furthermore, a significant positive correlation was also observed between 25-hydroxyvitamin D concentrations and measured miRNAs levels. A significant difference in observed miRNAs levels was also observed in patients with 25-hydroxyvitamin D insufficiency compared with patients with 25-hydroxyvitamin D concentration ≥20 ng/ml. And 1α,25(OH)_2_D_3_ differentially regulated miRNAs expression in dose- and time- manner *in vitro*. Lower expressions of miRNA-377, miRNA-342, miRNA-10a, miRNA-374b, miRNA-125a, and miRNA-410 were found in SLE patients. And severe vitamin D deficiency is associated with decreased observed miRNAs levels in SLE patients. A 25-hydroxyvitamin D concentration value <20 ng/ml is suggested as the “cut-off” for such immunological alterations in patients with SLE.

## INTRODUCTION

Systemic lupus erythematosus (SLE) is a systemic autoimmune disease with complex aberrant humoral and cellular immune responses [1–3]. Diverse T cell dysfunction, such as defective gene transcription and altered cytokine production, has been reported in patients with SLE [4]. Therefore, deregulated T cells could play an important role in lupus pathogenesis. MicroRNAs (miRNAs) are small conserved non-coding RNA molecules that regulate the expression of multiple target genes by targeting the 3’-untranslated region (UTR) of messenger RNAs (mRNAs), resulting in degradation or translational repression of mRNA. In the immune system, miRNA modulate both innate and adaptive immune responses [5]. Altered miRNA expression is implicated in the pathogenesis of many different autoimmune diseases [6–8]. Recently, several investigations have demonstrated that changed expression of miRNAs, including miR-21, miR-1246, miR-125a, and miR-155in T cells or peripheral blood mononuclear cells from patients with SLE is associated with innate immunity, DNA methylation, and inflammation [9–11]. The altered expression of miRNAs is found even in sera and urine from lupus patients, and is involved in the development of lupus nephritis [12]. In considering the extremely complex pathogenesis of immune dysfunction in SLE, it is possible that many more miRNAs might be involved in the immunopathogenesis of SLE. Thus, we hypothesized that aberrant expression of miRNAs in T cells from SLE patients would affect the downstream target molecule expressions that contribute to lupus pathogenesis.

In addition, more and more research regarding vitamin D has been published, especially on its immunoregulating effect, partly due to the expression of VDR on the surface of natural killer cells, antigen presenting cells, as well as B and T lymphocytes [13, 14]. It has also been demonstrated that vitamin D plays an important role on cytokines, such as reduction of Th1 cytokines such as interferon (IFN)-γ, IL-2, and tumor necrosis factor (TNF)-ɑproduction, inhibition of pro-inflammatory cytokines such as IL-6 and IL-17, and up-regulation of anti-inflammatory cytokines such as IL-4 and IL-10 [15, 16]. Both animal and epidemiological studies have suggested that vitamin D deficiency might predispose to some autoimmune diseases, such as SLE, systemic sclerosis (SSC), and rheumatoid arthritis (RA) [17–19]. It has already reported that vitamin D deficiency might have an effect on the phenotype of connective tissue diseases and low serum levels of vitamin D have been confirmed to be associated with higher disease activity in both adult and pediatric SLE patients [20]. In the animal model, the administration of vitamin D has been confirmed to ameliorate immune-mediated symptoms [21].

Whether vitamin D is associated with SLE risk has not been confirmed. Several detections have been performed to estimate the relationship between vitamin D level and SLE, including serum levels of 25(OH)D_3_, vitamin D intake, and variation in vitamin D related genes. However, examining vitamin D intake just account for a small portion of an individual’s vitamin D status since it does not reflect 25(OH)D_3_ produced as a result of exposure to sunlight nor the genetic regulation of vitamin D metabolism. Several genes/proteins play key role in converting 25(OH)D_3_ to 1α,25(OH)_2_D_3_(CYP27B1), degradation of 1α,25(OH)_2_D_3_ (CYP24A1) as well as enabling the action of 1α,25(OH)_2_D_3_.

There is increasing literature suggesting that the serum level of vitamin D and miRNAs expression are altered in SLE patients [22, 23]. Thus vitamin D and miRNAs might play an important role in the pathogenesis of SLE and its clinical manifestations. However, the association between the serum level of vitamin D and miRNAs expression involved in SLE patients has not been reported. The aim of our research was to evaluate the serum level of vitamin D and miRNAs expression in SLE patients and analyze the association between vitamin D, miRNAs expression and SLE.

## RESULTS

### Comparison of demographic characteristics and vitamin D levels between patients with SLE and healthy controls

A total of 90 individuals (42 SLE patients and 48 healthy controls) participated in this study. The demographic characteristics and vitamin D levels of patients and healthy controls are showed in Table 1 . The mean age of SLE patients was 36.19±11.72 years, while the healthy controls were 35.04±10.77 years. There was no significant difference in age, sex, BMI between patients and controls.

**Table 1:** Comparison of clinical parameters, miRNAs levels and 25-hydroxyvitamin D status between cases and controls

	SLE cases		Healthy controls		*P*
	Mean	SD	Mean	SD	
Total subjects (n)	42		48		
Male (n)	3		4		1.000
Female (n)	39		44		
Age (years)	36.19	11.72	35.04	10.77	0.629
BMI (kg/m^2^)	20.94	2.29	20.86	2.81	0.892
Vitamin D (nmol/L)	41.69	12.81	62.58	12.34	0.000
miRNA-410	0.0349	0.0262	0.1127	0.1429	0.001
miRNA-377	0.0007	0.0008	0.0046	0.0071	0.000
miRNA-342	73.3424	52.9918	649.02731	156.7059	0.001
miRNA-10a	0.0037	0.0041	0.0236	0.2987	0.000
miRNA-374b	3.9956	2.8982	37.0733	20.5389	0.000
miRNA-125a	0.0338	0.0224	0.1214	0.1799	0.002

**Figure 1:**
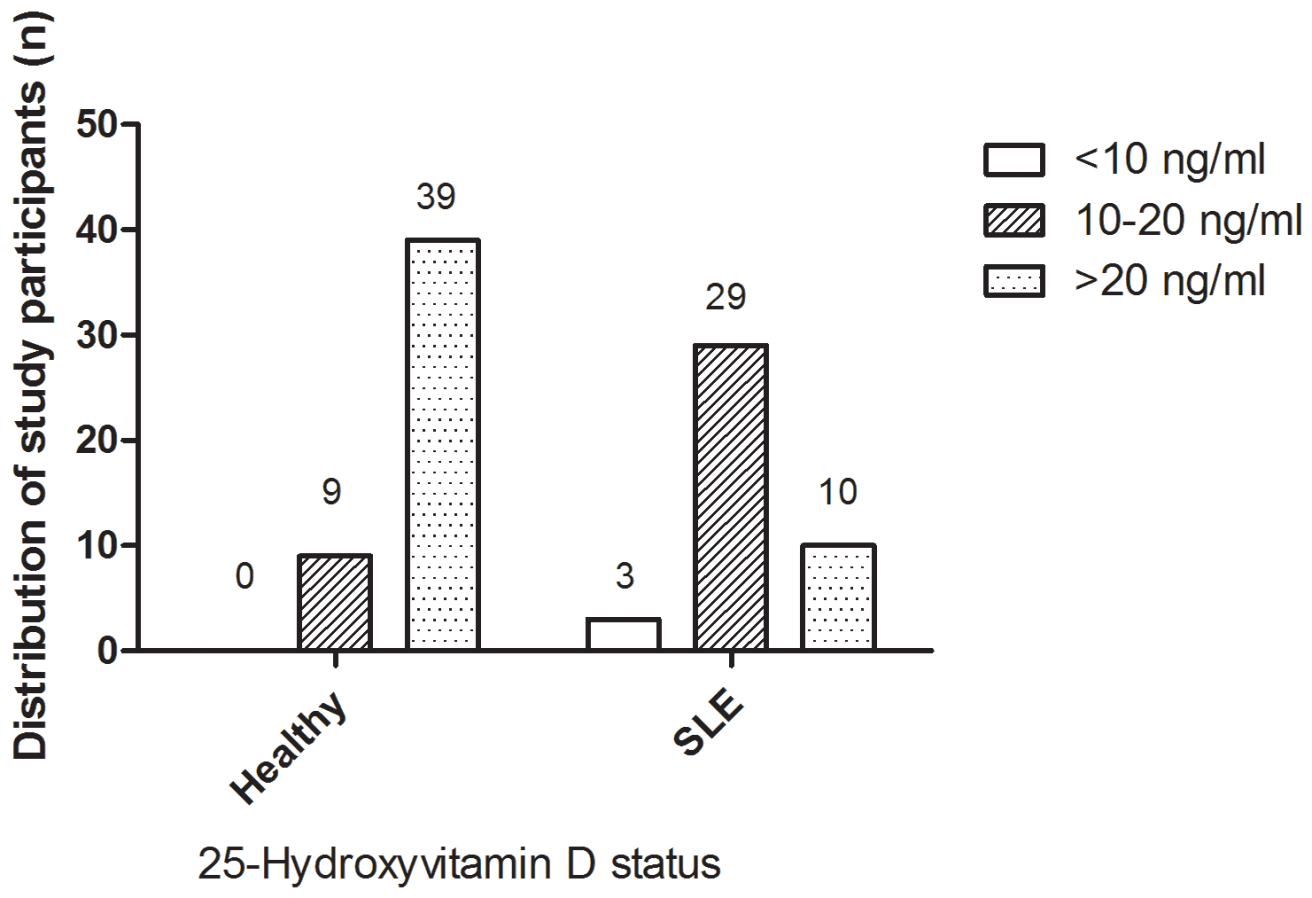
Distribution of study participants with respect to vitamin D status in SLE and healthy groups

The serum levels of vitamin D in SLE patients were significantly lower than that in healthy controls (16.68±5.13 *vs*. 25.03±4.94 ng/ml). There were 76.19% SLE patients who were classified as vitamin D insufficiency (<20 ng/ml), while 18.75% of healthy controls were vitamin D deficiency. The difference between SLE patients and healthy controls in vitamin D deficiency was statistically significant (χ^2^=29.798, *P*<0.001). Vitamin D deficiency (<10 ng/ml) was observed in 7.14% of SLE patients versus zero of healthy controls(*P*=0.098)(Figure 1).

### Clinical difference between patients with normal vitamin D levels and insufficient vitamin D levels

The clinical characteristics of the vitamin D insufficiency patients (30 cases) and vitamin D normal patients (12 cases) were compared and the data were showed in Table 2 . The results showed that there were no significant difference in the SLE symptoms and clinical features, including SLEDAI scores, disorder rash, renal disorder, photosensitivity, oral ulcers, serositis, anti-nuclear antibody, and anti-dsDNA. The ratio of malar rash and arthritisin vitamin D insufficiency patients was higher than those in patients without vitamin D insufficiency, and the difference were statistically significant.

**Table 2:** Comparison of clinical data between patients with vitamin D insufficiency and patients with normal vitamin D levels

Clinical data	Serum levels of vitamin D		*P*
	Insufficient	Normal	
Count	32	10	
SLEDAI score(M±Q)	10±2	8±3.5	0.512
Malar rash(+/-)	24/8	3/7	0.027
Discoid rash(+/-)	10/22	4/6	0.898
Renal disorder (+/-)	11/21	4/6	1.000
Photosensitivity(+/-)	14/18	4/6	1.000
Oral ulcers(+/-)	9/23	3/7	1.000
Arthritis(+/-)	20/12	2/8	0.047
Serositis(+/-)	14/18	5/5	1.000
Anti-nuclear antibody (+/-)	22/10	5/5	0.483
Anti-dsDNA(+/-)	11/21	4/6	1.000

### Identification and verification of differential expression of miRNAs in T cells from patients with SLE and controls

**Figure 2:**
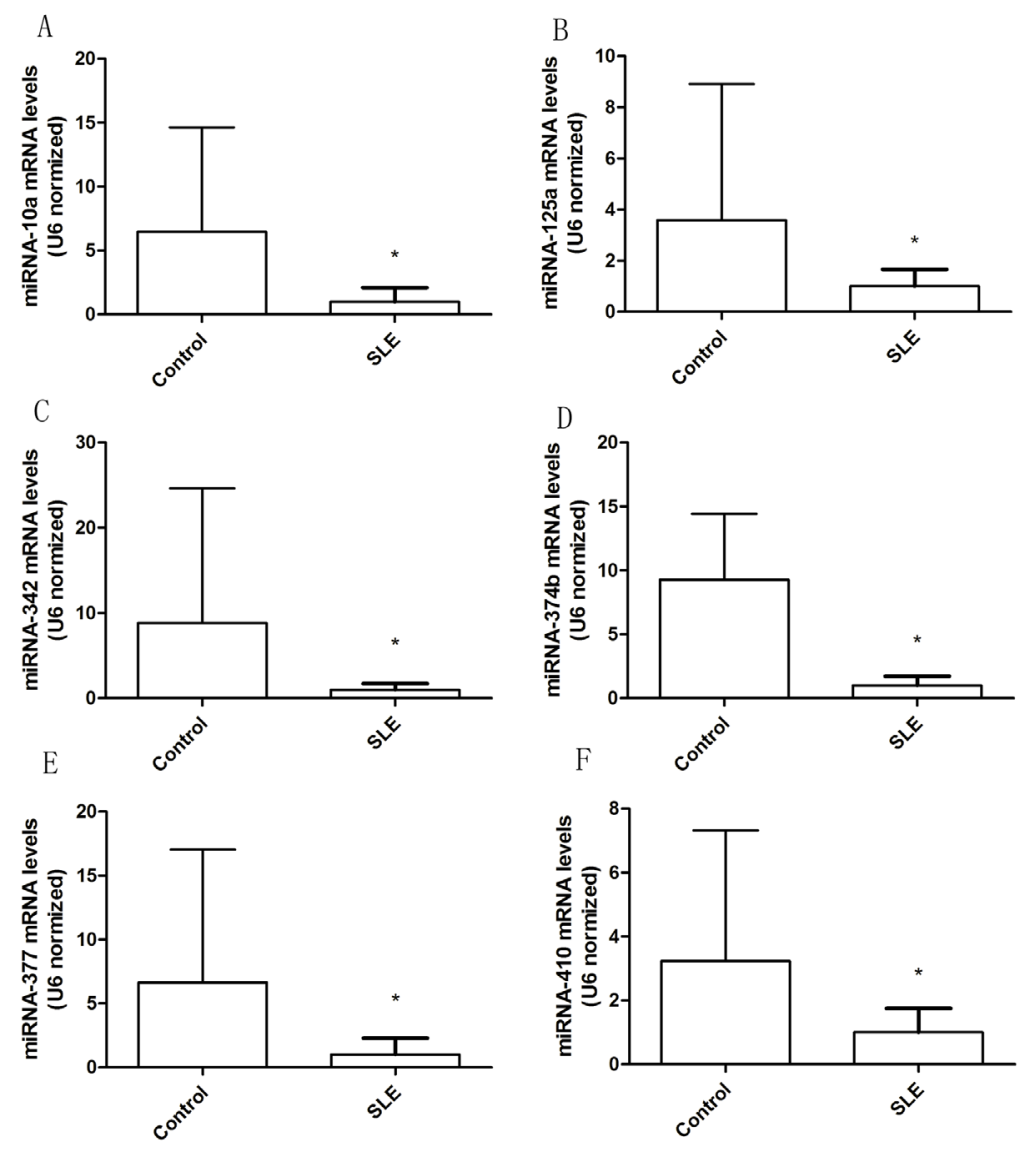
Altered expression of miRNA in T cells from patients with SLE The six abnormally expressed miRNAs in T cells were validated by real-time PCR from 42 SLE patients and 48 healthy controls. **(A)** miRNA-10a levels in T cells from SLE and control groups. **(B)** miRNA-125a levels in T cells from SLE and control groups. **(C)** miRNA-342 levels in T cells from SLE and control groups. **(D)** miRNA-374b levels in T cells from SLE and control groups. **(E)** miRNA-377 levels in T cells from SLE and control groups. **(F)** miRNA-410 levels in T cells from SLE and control groups. The error bars represents the standard deviation (SD). * *p*<0.05 *vs* con.

To identify miRNAs that were expressed potentially differentially in SLE T cells, we analyzed the expression levels of miRNA-377, miRNA-342, miRNA-10a, miRNA-374b, miRNA-125a, and miRNA-410 in T cells from 42 patients and 48 controls. And the results suggested that expression of these sixmiRNAsin T cells were significantly different between SLE patients and healthy controls (Figure 2). The mean expression of miRNA-377 (*P*=0.001), miRNA-342 (*P*=0.001), miRNA-10a (*P*=0.000), miRNA-374b (*P*=0.000), miRNA-125a (*P*=0.002), and miRNA-410 (*P*=0.001)were significantly lower in cases than that in controls.

### Correlation of serum levels of vitamin D and miRNAs expression in T cells from SLE patients and controls

**Figure 3:**
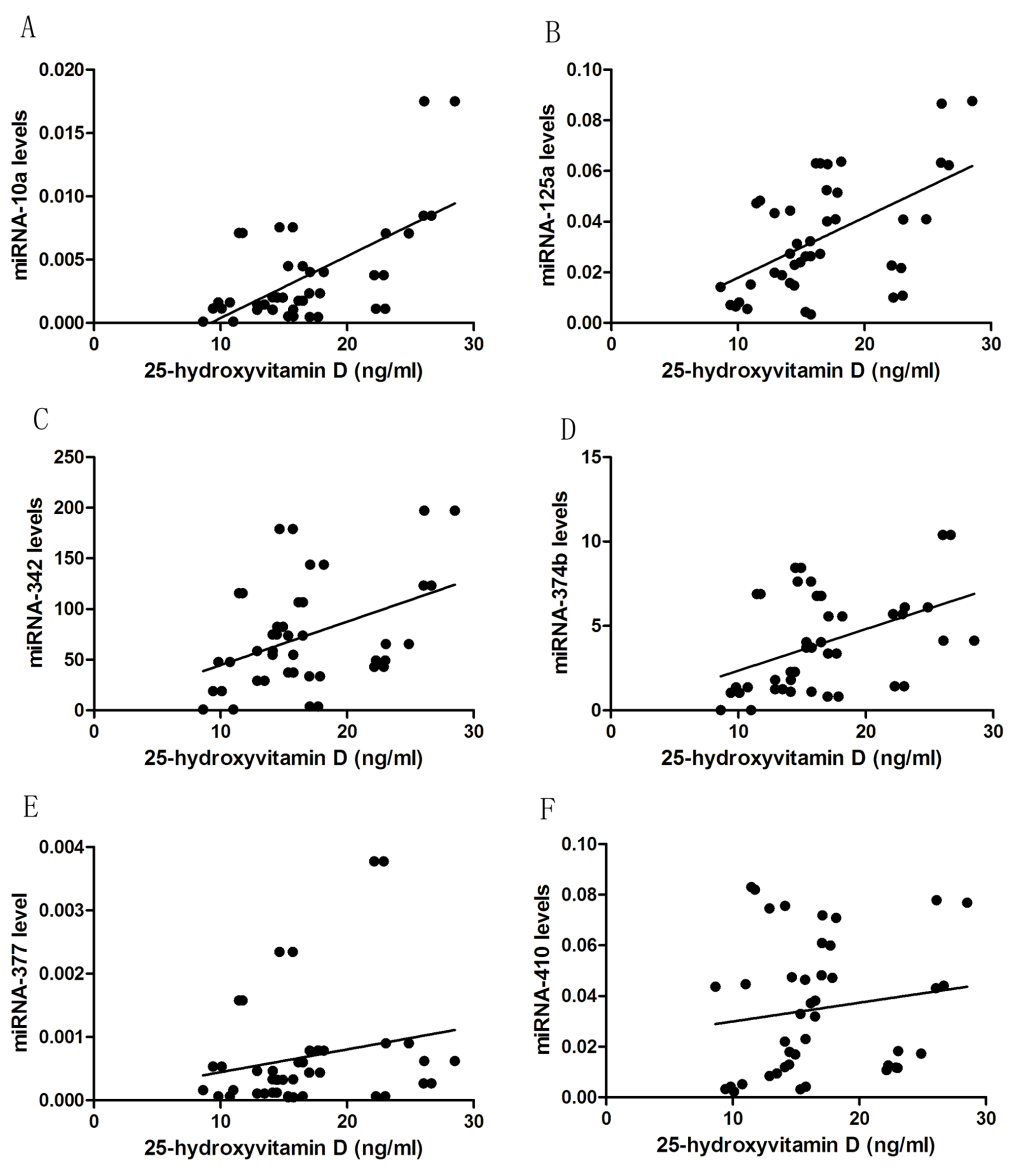
The correlation between 1α,25(OH)_2_D_3_ levels and miRNAs expression in T cells from SLE patients **(A)** The correlation between 1α,25(OH)_2_D_3_ levels and miRNA-10a expression in T cells from SLE patients. **(B)** The correlation between 1α,25(OH)_2_D_3_ levels and miRNA-125a expression in T cells from SLE patients. **(C)** The correlation between 1α,25(OH)_2_D_3_ levels and miRNA-342 expression in T cells from SLE patients. **(D)** The correlation between 1α,25(OH)_2_D_3_ levels and miRNA-374b expression in T cells from SLE patients. **(E)** The correlation between 1α,25(OH)_2_D_3_ levels and miRNA-377 expression in T cells from SLE patients. **(F)** The correlation between 1α,25(OH)_2_D_3_ levels and miRNA-410 expression in T cells from SLE patients. There were statistically significant correlation between the relative miRNAs expressions of miRNA-377 (r_s_=0.473, *P*<0.001), miRNA-342 (r_s_=0.634, *P*<0.001), miRNA-10a (r_s_=0.649, *P*<0.001), miRNA-374b (r_s_=0.682, *P*<0.001), miRNA-125a (r_s_=0.455, *P*<0.001), and miRNA-410 (r_s_=0.347, *P*=0.001) and 1α,25(OH)_2_D_3_ levels.

In both cases and controls, 25-hydroxyvitamin D concentration was found to be significantly positively correlated with miRNA-377 (*r*_*s*_=0.473, *P*<0.001), miRNA-342 (*r*_*s*_=0.634, *P*<0.001), miRNA-10a (*r*_*s*_=0.649, *P*<0.001), miRNA-374b (*r*_*s*_=0.682, *P*<0.001), miRNA-125a (*r*_*s*_=0.455, *P*<0.001), and miRNA-410 (*r*_*s*_=0.347, *P*=0.001) (Figure 3). Independent correlation analysis revealed a significant positive correlation between vitamin D and miRNAs expression in the study participants, except for miRNA-410 and miRNA-377, which exhibited non-significant correlation independently in patients, and the six miRNAs expression were non-significant correlation independently in controls (Table 3).Patients with 25-hydroxyvitamin D concentration<20 ng/ml had significantly lower expression of miRNAs than Patients with normal 25-hydroxyvitamin D levels, including miRNA-10a (*P*=0.022), miRNA-374b (*P*=0.043) and miRNA-125a (*P*=0.034) (Table 4).

**Table 3:** Correlation between vitamin D and miRNAs expression in cases, controls and total population.

Vitamin D	SLE patients			Healthy controls			Total		
	*n*	*r*_*s*_	*p*	*n*	*r*_*s*_	*p*	*n*	*r*_*s*_	*p*
miRNA-377	42	0.262	0.093	48	0.280	0.054	90	0.473	0.000
miRNA-342	42	0.365	0.018	48	0.223	0.127	90	0.634	0.000
miRNA-10a	42	0.462	0.002	48	0.181	0.217	90	0.649	0.000
miRNA-374b	42	0.439	0.004	48	0.256	0.080	90	0.682	0.000
miRNA-125a	42	0.525	0.000	48	0.152	0.302	90	0.455	0.000
miRNA-410	42	0.237	0.131	48	0.266	0.067	90	0.347	0.001

**Table 4:** Comparison of miRNAs levels between SLE patients with different vitamin D status

	Insufficiency		Normal		*P*
	Mean	SD	Mean	SD	
miRNA-410	0.0349	0.0262	0.1127	0.1429	0.001
miRNA-377	0.0007	0.0008	0.0046	0.0071	0.000
miRNA-342	73.3424	52.9918	649.02731	156.7059	0.001
miRNA-10a	0.0037	0.0041	0.0236	0.2987	0.000
miRNA-374b	3.9956	2.8982	37.0733	20.5389	0.000
miRNA-125a	0.0338	0.0224	0.1214	0.1799	0.002

### Different expression of VDR, CYP27B1, and CYP24A1 mRNA in T cells from patients and controls

**Figure 4:**
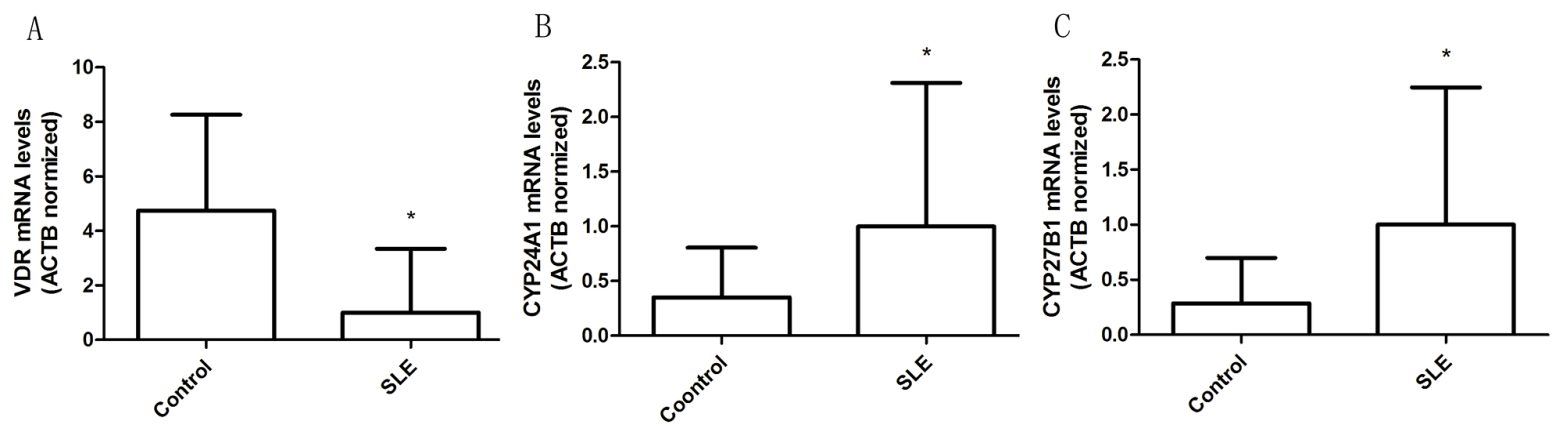
Altered expression of vitamin D receptor signaling protein in T cells from patients with SLE **(A)** VDR mRNA levels in T cells from SLE and control groups. **(B)** CYP24A1 mRNA levels in T cells from SLE and control groups. **(C)** CYP27B1 mRNA levels in T cells from SLE and control groups. The error bars represents the standard deviation (SD). * *p*<0.05.

Due to the lower levels of vitamin D in SLE patients than that in healthy controls, we also examined integrity of vitamin D signaling in the T cells. We assessed the expression and induction of VDR, CYP24A1 and CYP27B1 within qRT-PCR. Results showed that VDR mRNA expressions in T cells of SLE patients were significantly lower than those in controls, but CYP24A1 and CYP27B1 mRNA levels were significantly increased (Figure 4).

### 1α,25(OH)_2_D_3_ differentially regulates miRNAs expression in cultured T cells from SLE patients

**Figure 5:**
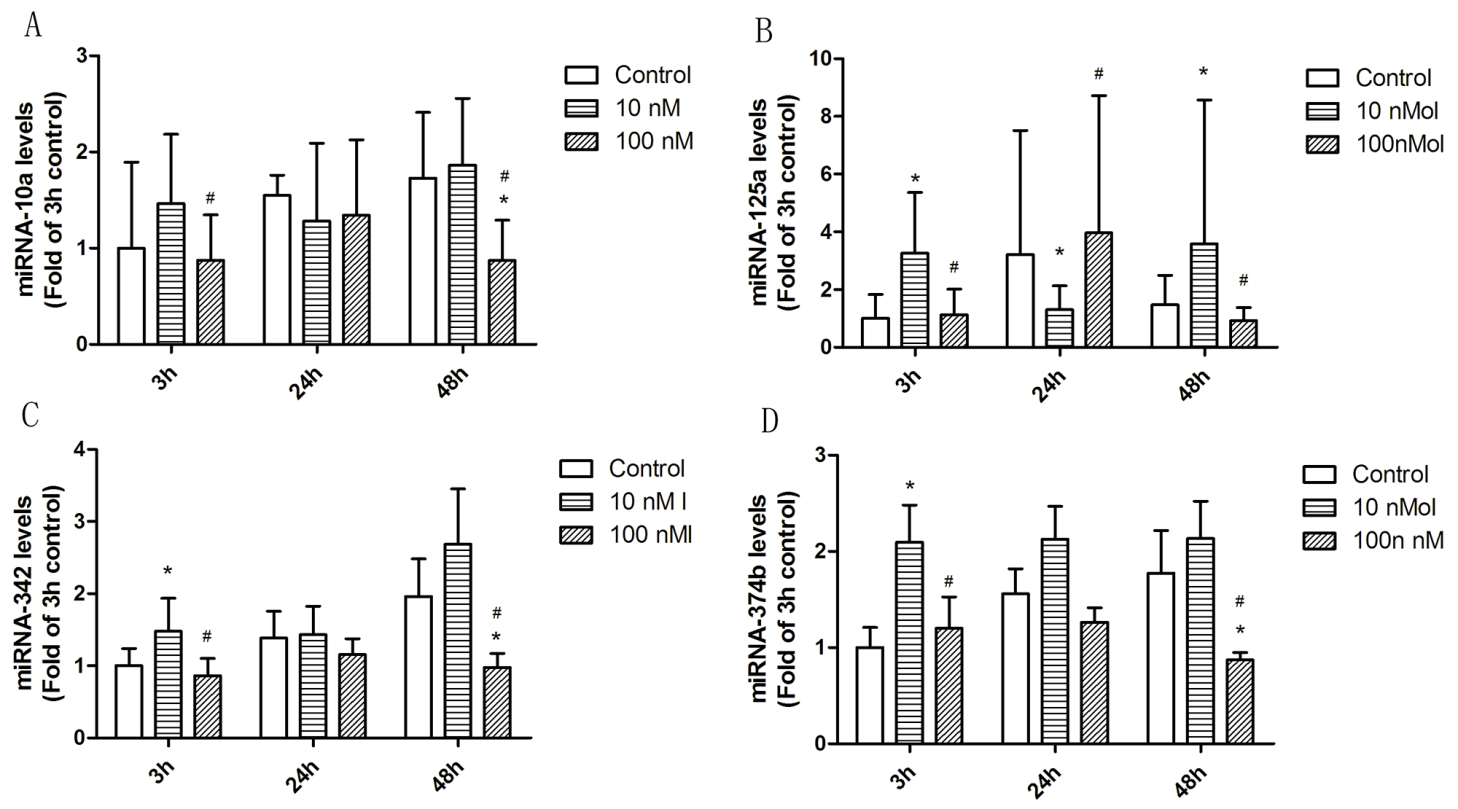
The effect of 1α,25(OH)_2_D_3_ on miRNA expression in T cells from SLE patients (**A**) The effect of 1α,25(OH)_2_D_3_ on miRNA-10a expression in T cells from SLE patients. (**B**) The effect of 1α,25(OH)_2_D_3_ on miRNA-125a expression in T cells from SLE patients. (**C**) The effect of 1α,25(OH)_2_D_3_ on miRNA-342 expression in T cells from SLE patients. (**D**) The effect of 1α,25(OH)_2_D_3_ on miRNA-374b expression in T cells from SLE patients. T cells were plated as described in methods and treated with 10 nM and 100 nM 1α,25(OH)_2_D_3_ for 3h, 24h and 48h. The error bars represents the standard deviation (SD). n=16 for each experiment. * *p*<0.05 *vs* con; # *p*<0.05 100 nM *vs* 10 nM treatment.

To investigate the impact of 1ɑ,25(OH)_2_D_3_ on the expression of miRNAs, T cells from SLE patients were treated with 10nM, and 100nM for 3h, 24h, and 48h and the expression of miRNA-342, miRNA-10a, miRNA-374b, and miRNA-125a were examined by qRT-PCR assays [25]. Within T cells, 1ɑ,25(OH)_2_D_3_ markedly regulated miRNAs expression as compared to ETOH control, and the regulation showed dynamic changes at different time (Figure 5).

**Figure 6:**
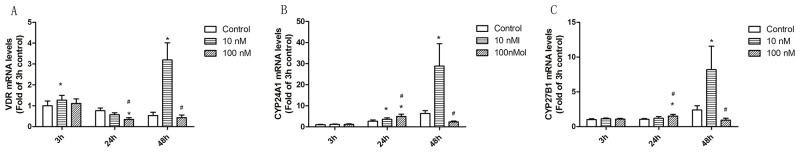
The effect of 1α,25(OH)_2_D_3_ on mRNA expression in T cells from SLE patients (**A**) The effect of 1α,25(OH)_2_D_3_ on VDR mRNA expression in T cells from SLE patients. (**B**) The effect of 1α,25(OH)_2_D_3_ on CYP24A1 mRNA expression in T cells from SLE patients. (**C**) The effect of 1α,25(OH)_2_D_3_ on CYP27B1 mRNA expression in T cells from SLE patients. T cells were plated as described in methods and treated with 10 nM and 100 nM1α,25(OH)_2_D_3_ for 3h, 24h and 48h. The error bars represents the standard deviation (SD). n=16 for each experiment. * *p*<0.05 *vs* con; # *p*<0.05 100 nM *vs* 10 nM treatment.

**Figure 7:**
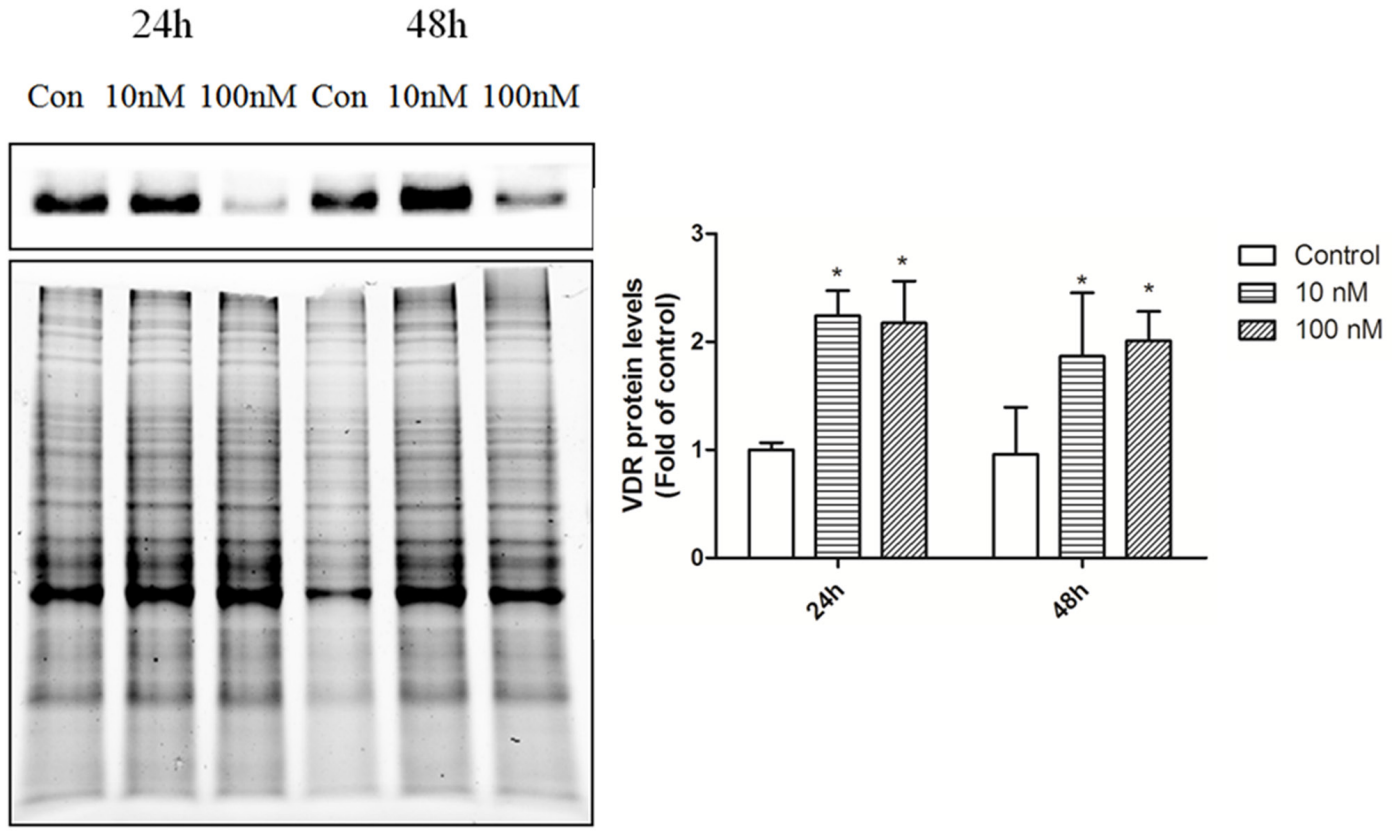
VDR protein expression in T cells from SLE patients T cells were treated with control ETOH or 10 nM, 100 nM 1α,25(OH)_2_D_3_for 24h and 48h. VDR protein expression was examined by immunoblot analysis. The error bars represents the standard deviation (SD). n=16 for each experiment. * *p*<0.05 *vs* con; # *p*<0.05 100 nM *vs* 10 nM treatment.

To investigate the potential role of 1ɑ,25(OH)_2_D_3_in the regulation of miRNAs, we also examined integrity of vitamin D signaling in the T cells. We assessed the expression and induction of VDR, CYP24A1 and CYP27B1, which mediates most of the activities of 1ɑ,25(OH)_2_D_3_ and are also transcriptionally regulated by themselves. T cells were treated with vehicle control and 10nM, and 100nM for 3h, 24h, and 48h and VDR, CYP24A1 and CYP27B1 mRNA/protein expression were evaluated by qRT-PCR and immunoblot analysis. 1ɑ,25(OH)_2_D_3_ induced VDR, CYP24A1and CYP27B1 expression in a dose dependent manner in T cells (Figures 6 and 7). These results indicate that T cells have functional 1ɑ,25(OH)_2_D_3_ signaling.

## DISCUSSION

It has recently become evident that miRNA plays an important role in immune homeostasis and aberrant expression of certain miRNAs, which is thought to contribute to the initiation and progression of autoimmunity [26, 27]. Altered miRNA expression has been reported in the pathology of SLE in several studies [28, 29]. Despite the abundance of studies showing the involvement of miRNAs in SLE, the precise role of dysregulated miRNAs levels in SLE remains to be determined. miRNAs are a group of newly discovered non-coding RNAs of 22 nucleotides that are widely present in tissue, plasma, and other body fluids. They can cause the degradation of mRNA or suppress gene translation by interacting with the 3’UTR of targeted mRNA. Recent studies have shown that alteration of the expression of miRNAs is closely associated with autoimmune disease by considering that aberrant expression of miRNA 21 and miRNA 126 is closely associated with initiation and development of dysfunction of T cells [23, 30]. Furthermore, through the function predication and analysis with TargetScan database, we found miRNA 10a, miRNA125a, miRNA 342, miRNA 374b, miRNA 377, and miRNA 410 might be associated with vitamin D receptor and vitamin D signaling pathway. However, to date, the expression of miRNA 10a, miRNA125a, miRNA 342, miRNA 374b, miRNA 377, and miRNA 410 and their function in autoimmune diseases, such as SLE, and the association with vitamin D status, have rarely been reported.

In the present study, patients with SLE served as a model for significant immunological defects, and the concentrations of miRNAs were obviously altered in T cells from SLE patients. Our results were similar to those reported by Liu et al., who showed non-random significant alteration of miRNAs expression such as miRNA-126 and miRNA-133 in patients with SLE [31].

The variables known to influence miRNAs expression such as BMI, age, and duration of SLE were similar between the two groups in this study. Interestingly, there was a positive correlation between those six miRNAs and vitamin D concentration. Similarly, results from another study have also shown that 1,25-dihydroxyvitamin D status was associated with the expression of miRNAs expression in patients with coronary artery disease [31]. However, several findings reported in healthy women with variable cut-off values of 25-hydroxyvitamin D were in contrast to the above findings and failed to demonstrate any relationship with miRNAs expression [32, 33]. The levels of miRNAs expression are influenced by several physiological factors, and we proposed that vitamin D could be one of them. There is no definite cut-off value defined for miRNAs expression that could help to differentiate healthy and diseased status or indicating the severity of the disease; thus, to study the association between vitamin D status and miRNAs levels, a correlation analysis using Spearman’s correlation was carried out in the present study.

Vitamin D deficiency and its various implications have been investigated in patients with SLE. Previous studies have suggested that 1,25-dihydroxyvitamin D regulates both innate and adaptive immunity, potentiating the innate response, which has been shown to precede the development of autoimmunity in SLE, but reducing adaptive immunity, including reduced T cell activation and working in conjunction with glucocorticoids on the inhibition of lymphocyte proliferation [34–38]. Vitamin D deficiency seems to play a role in increasing B cell activation and therefore autoantibody production, and long-term supplementation with vitamin D increased the number of T-regulator cells in individuals with SLE [39, 40]. 1,25-dihydroxyvitamin D has inhibitory effects on many of the immunological response pathways associated with SLE, including the proliferation of B cells, antibody production and altered inflammatory and regulatory pathways [41, 42]. In addition, 1,25-dihydroxyvitamin D has been shown to downregulate pro-inflammatory cytokines in macrophages by decreasing aromatase activity [43]. Vitamin D deficiency, and therefore reduced 1,25-dihydroxyvitamin D levels, may result in an increased risk of transitioning to SLE through these pathways. In the present study, vitamin D insufficiency was more common in patients with SLE than in healthy controls. We have found vitamin D concentration of 20 ng/ml might be a possible risk factor for SLE with a proposition that it might result in miRNA expression dysregulation. In this study, we choose this cut-off value to assess the difference in miRNA status defined by miRNAs levels at this risk point and found that there was significant difference in the levels of miRNA-10a, miRNA-374b, and miRNA-125a in patients with vitamin D insufficiency (serum 25-hydroxyvitamin D concentration<20 ng/ml) in comparison with patients with serum 25-hydroxyvitamin D concentration≥20 ng/ml, which substantiated our previous finding.

Vitamin D and miRNAs are intrinsic factors, whereas SLE is a systemic autoimmune disease that influences miRNAs expression in the host. We tried to identify vitamin D insufficiency as an additional factor contributing to miRNAs dysfunction in SLE group. The positive association between vitamin D and miRNAs levels in SLE patients was found to be disrupted. The cross-sectional design is a limitation and therefore we performed an*invitro*experiment on T cells response to vitamin D stimulation and miRNAsexpression.The results wereinaccordance with the case-control study findings and demonstrated increased expression of miRNA-377, miRNA-342, miRNA-10a, miRNA-374b, miRNA-125a, and miRNA-410 in cultured T cells from SLE patients compared with normal T cells with supplement of vitamin D.In addition, we also found activation of vitamin D signaling such as VDR, CYP24A1, and CYP27B1, which mediates most of the activities of 1α,25(OH)_2_D_3_ in the T cells [44].

The results of this study showed that altered responses occur as a consequence of vitamin D deficiency in patients with SLE and also served to redefine the cut-off value of 25-hydroxyvitamin D deficiency for healthy and diseased populations. Based on these results, we suggested 1 25-hydroxyvitamin D concentration value<20 ng/ml to be the cut-off for unfavorable immunological alteration. Studies including various other immunological parameters would provide evidence for this distinctive hypothesis and these results.

In conclusion, vitamin D deficiency reduced expression of miRNA-342, miRNA-10a, miRNA-374b, and miRNA-125a in T cells of patients with SLE, particularly when its serum concentration was very low. And vitamin D supplement alter those miRNAs expression in isolated T cells from patients with SLE. Therefore, we suggest a 25-hydroxyvitamin D concentration value <20 ng/ml as the “cut-off” for unfavorable immunological alterations in patients with SLE. Furthermore, we found a typical interplay of several miRNAs expression and vitamin D to have clinical implications for patients with SLE.

## MATERIALS AND METHODS

### Patients and controls

During the years 2014-2015, forty-two new cases diagnosed by SLE in total satisfying the 1982 American College of Rheumatology (ACR) revised criteria for the classification of SLE have been recruited, and 48 healthy volunteers served as a control group. Each participant signed an informed consent approved by the local Internal Review Board and Ethnicity Committee of Anhui Medical University. Demographic and clinical data of the SLE patients were recorded. Blood samples were collected at least 18h after the last dose of immune-suppressants in order to minimize drug effects in the *in-vitro* studies. All procedures performed in this study involving human participants were in accordance with the ethics standards of the institutional and national research committee and with the 1964 Helsinki Declaration and its later amendments or comparable ethics standards.

### Reagents

1ɑ,25(OH)_2_D_3_ (Abcam, USA) was reconstituted in 100% ethanol and stored, protected from light, at -20°C. Anti-vitamin D receptor (VDR) was purchased from Santa Cruz Biotechnology (Santa Cruz, CA). IMag Cell Separation System was from MiltenyiBiotec, Germany. The miRNeasy kit for RNA isolation was from Qiaqen, USA. 1×SYBR Master Mix was from GeneCopoeia, USA. The qRT-PCR kit was also from GeneCopoeia, USA. Magnetic separation was from MiltenyiBiotec, Germany. The primers of miRNA-10a (ID: hsmq-0760), miRNA-125a (ID: hsmq-0483), miRNA-342 (ID: hsmq-0481), miRNA-374b-5p (ID: hsmq-0585), miRNA-377 (ID: hsmq-0255), miRNA-410 (ID:hsmq-0161), U6 (ID:hsnRNAU6), and VDR (ID:Hs-QRP-40488), CYP27B1 (ID:Hs-QRP-30404), CYP24A1 (ID:Hs-QRP-30402), and β-actin (ID:Hs-QRP-20056) were designed by GeneCopoeia USA.

### Cell isolation and culture

Peripheral blood mononuclear cells (PBMCs) of 16 SLE patients were freshly isolated by Ficoll-Histopaque density gradient centrifugation of heparinized venous blood. CD3^+^ T lymphocytes were obtained by magnetic separation. Freshly isolated T lymphocytes were seeded in 6-well tissue culture plates, and grown in RPMI-1640 medium supplemented with 10% FBS, 100 U/ml penicillin and 100μg/ml streptomycin. Cells were maintained at 37°C in a humidified atmosphere of 95% air/5% CO_2_. For all the experiments performed in this study, isolated T cells were plated at a density of 1×10^6^ cells/ml for 3h, 24h, and 48h prior to treatment with different doses of 1α,25(OH)_2_D_3_. The steroids were dissolved in ethanol, and control cells were treated with the same volume of vehicle.

### Serum sample collection and vitamin D level measurement

Serum samples were obtained from 3 ml whole blood samples of patients and controls by using centrifugation (1500g for 10 min). All serum samples were frozen at -80°C until required for analysis. Serum 25(OH)D_3_ levels were measured with 25-hydroxy vitamin D Kit (IDS,UK) at the same laboratory. Normal levels of vitamin D were defined as concentrations>20 ng/ml. Serum levels between 10 and 20 ng/ml were classified as vitamin D insufficiency, while serum levels of vitamin D <10 ng/ml were classified as vitamin D deficiency [24].

### Preparation of RNA from T cells

Blood obtained from SLE patients and healthy controls was layered over a Ficoll-Hypaque density gradient solution. After centrifugation (250-300g) at room temperature for 25 min, mononuclear cells were puried further using anti-human CD3 magnetic particles using an IMag Cell Separation System. Total RNA, including miRNA, was extracted from the purified T cells using the miRNeasy kit according to the manufacture’s protocol. The concentration of RNA was quantified using a NanoDrop Spectrophotometer.

### Reverse transcription of RNAs

All the extracted RNAs were converted into corresponding cDNAs by All-in-One^TM^ First-Strand cDNA SynthesisKit(GeneCopoeia,USA) according to the manufacture’s protocol. Briefly, a final 25ulreaction mixture containing 1ul2.5U/ul Poly A Polymerase, 1ulRTase Mix, 5ul 5×PAP/RT Buffer, and 2ugtotal RNA were used for the RT reaction. Then mixing the prepared reactioningentiy.Incubating the mixture at 85°C for 5 min to inactivate the enzyme following incubation at 37°C for 60 min after a brief centrifugation.

### Measurement of miRNA expression by real-time PCR

A real-time PCR-based method was used to quantify the expression levels of miRNAs expression within the protocol of All-in-One^TM^miRNA qRT-PCR Detection Kit (GeneCopoeia,USA). Each PCR reaction contained 10ul 2×All-in-One^TM^qPCR Mix, 2ul All-in-One^TM^miRNA qPCR Primer,2ul Universal Adaptor PCR Primer,2ul First-stand cDNA 0.4ul 50×ROX Reference Dye and 3.6ul ddH_2_O. All reactions were performed in duplicate on an ABI ViiA^TM^7 real-time PCR system. The conditions for quantitative PCR were 95°C for 10 min, followed by 42 cycles of 95°C for 10 s , 60°C for 20 s and 72°C for 20 s. Expression of the U6 small nuclear RNA was used as an endogenous control for data normalization. The threshold cycle (Ct) is defined as the cycle number at which the fluorescence intensity change crosses the average background level of the fluorescence signal. In the initial screening studies, the normalized miRNA level was defined by the equation with global median normalization before further analysis. For the analysis of individual miRNAexpression, the value of each Ct was first normalized by the U6 small nuclear RNA and then the normalized miRNA level was defined by the equation.

### Measurement of VDR, CYP27B1 and CYP24A1 mRNA expression by real-time PCR

The expression level of VDR,CYP27B1 and CYP24A1 mRNA were quantified by real-time PCR using AceQTMqPCR SYBR Green Kit (Vazyme Biotech,China) on an ABI ViiA^TM^7 real-time PCR system.The conditions for quantitative PCR were 95°Cfor 10 min, followed by 40 cycles of 95°C for 10 s , 60°C for 20 s and 60°C for 20 s. Expression ofβ-actin mRNA was used as endogenous control for data normalization.

### Protein detection

Protein samples were run on Bio-Rad TGX Stain-Free precast gels and visualized on the UV setting in the BioRad Chemidoc MP Imaging System to estimate total protein per lane. Blocking and antibody incubations were done in NAP blocker^TM^ (G-Biosciences). The mouse anti-VDR (Abcam) was used 1:10,000 and incubations were done overnight at 4°C. For detection, the blots were incubated overnight at 4 °C with anti-mouse biotin (Jackson Laboratories) followed by anti-mouse streptavidin-HRP (Vectastain Elite ABC system, Vector Laboratories). Molecular weight markers are Precision Plus Protein Kaleidoscope Standards (BioRad). Protein detection was achieved with femtoCHROMO^TM^–HRP (G-Biosciences) and visualized using the colorimetric setting in the BioRad Chemidoc MP Imaging System.

### Statistical analysis

Data are presented as means and standard deviations or as means with their standard errors unless otherwise indicated. Continuous variables that were not normally distributed were logarithmically transformed before analysis. Statistical analysis was conducted using SPSS 18.0. Parametric and non-parametric tests were used wherever applicable. Correlation analysis (Spearman) was carried out to determine the association between these miRNAs expression and 25-hydroxyvitamin D status in the study population.
